# Melatonin and Pancreatic Islets: Interrelationships between Melatonin, Insulin and Glucagon

**DOI:** 10.3390/ijms14046981

**Published:** 2013-03-27

**Authors:** Elmar Peschke, Ina Bähr, Eckhard Mühlbauer

**Affiliations:** 1 Saxon Academy of Sciences, Leipzig 04107, Germany; E-Mail: eckhard.muehlbauer@uk-halle.de; 2 Institute of Anatomy and Cell Biology, Martin Luther University Halle-Wittenberg, Halle (Saale) 06108, Germany; E-Mail: ina.baehr@medizin.uni-halle.de

**Keywords:** melatonin, MT1 and MT2 receptors, insulin, glucagon, type 1 and type 2 diabetes

## Abstract

The pineal hormone melatonin exerts its influence in the periphery through activation of two specific trans-membrane receptors: MT1 and MT2. Both isoforms are expressed in the islet of Langerhans and are involved in the modulation of insulin secretion from β-cells and in glucagon secretion from α-cells. De-synchrony of receptor signaling may lead to the development of type 2 diabetes. This notion has recently been supported by genome-wide association studies identifying particularly the MT2 as a risk factor for this rapidly spreading metabolic disturbance. Since melatonin is secreted in a clearly diurnal fashion, it is safe to assume that it also has a diurnal impact on the blood-glucose-regulating function of the islet. This factor has hitherto been underestimated; the disruption of diurnal signaling within the islet may be one of the most important mechanisms leading to metabolic disturbances. The study of melatonin–insulin interactions in diabetic rat models has revealed an inverse relationship: an increase in melatonin levels leads to a down-regulation of insulin secretion and *vice versa*. Elucidation of the possible inverse interrelationship in man may open new avenues in the therapy of diabetes.

## 1. Introduction, Historical Aspects

Nearly 75 years ago, one of the first references to the functional connection between the pineal gland and carbohydrate metabolism was made by the Romanian Constantin I. Parhon in a short abstract [1]. In the following years, a controversial discussion was carried out in many publications on the importance of pineal extracts in glucose metabolism [2–7]. Even later, after the isolation [8] and identification of the molecular structure of melatonin (5-methoxy-*N*-acetyltryptamine) by Aron Lerner and colleagues [9] this discussion continued. The results of the Romanian group can be summarized as follows: a pineal peptide, named “pinealin”, was described as being similar to insulin in that it displays hypoglycemic, anabolic, anti-cholesterinemic and glomerulotrophic characteristics [5]. Pinealin increases glucose tolerance, whereas pinealectomy decreases insulin secretion and glucose tolerance [6,10,11]. These results are in accordance with later findings of the Spanish group of Blásques *et al.*[12–14] who indicated that pinealectomy or bilateral sympathetic denervation of the pineal gland significantly reduces insulin levels and increases blood glucose [15–18]. These effects could be counteracted by melatonin application [19]. In this context, later observations are also important which indicate that melatonin suppresses the onset of type 1 diabetes, whereas pinealectomy promotes the development of this metabolic disease [20,21].

In contrast to the above-mentioned investigations, it has very recently been published that high melatonin levels, due to blindness [22] or through exogenous application of melatonin, raise blood glucose levels [23], whereas a decrease in blood glucose [24,25] and an increase in insulin levels are observed after pinealectomy [26–29]. However, most authors agree that the pineal gland has a suppressive effect on the activity of the pancreatic insulin-producing β-cell, because melatonin reduces insulin levels [30–32] and glucose tolerance in rats [33,34]. Based on the results that an increased insulin level exerts an inhibitory effect on the melatonin synthesis of the pineal gland [35,36], a functional antagonism between insulin and melatonin has to be assumed. This antagonism is in line with the fact that, in man, the low insulin levels at night and high levels during the day coincide with elevated nocturnal melatonin concentrations and reduced levels during the day [37]. In addition, diabetic patients are largely devoid of a circadian melatonin rhythm [38,39]. Altogether, the cited findings clearly indicate that melatonin-insulin interactions, which are modulated and regulated by catecholamines, particularly norepinephrine [40–46], play an important role in glucose metabolism.

Contradictory results pervade the cited literature concerning the modulating effects of melatonin on the pancreatic β-cell as well as on their insulin secretion. A functional connection between melatonin and several features of carbohydrate metabolism has been convincingly described. However, there are scientific investigations which deny an influence of melatonin on β-cells and insulin secretion. For example, in *in vitro* experiments [47,48] and research on rats [49,50] melatonin was not shown to have an inhibitory influence on glucose-induced insulin secretion.

Prior to the above-cited publications, no information had been available as to whether the MT1 receptor was located on the β-cell. To examine this aspect, a glucose-responsive, insulin-producing rat insulinoma cell line (INS-1) was employed. Compared to earlier results on islets, the competitive receptor antagonist luzindole diminished the insulin-decreasing effect of melatonin. In addition, RT-PCR experiments using specific primers for the rat melatonin receptor MT1 showed that MT1 mRNA is also expressed in INS-1 cells, as well as in islets and whole rat pancreas [51,52].

## 2. Membrane Melatonin Receptors of the Pancreatic β-Cell

### 2.1. Function and Signaling of Mammalian Melatonin Membrane Receptors

Although the indoleamine melatonin is considered a lipophilic molecule, there is general agreement that most, if not all, of the previously reported effects are mediated, in mammals, by the membrane receptors MT1 and MT2 [53,54]. A third member of this class of G-protein-coupled receptors (GPCRs), Mel1c, is known to be functionally present in low vertebrates, reptiles and birds. Only recently has it become clear that the well-known melatonin-related orphan receptor (G-protein-coupled receptor 50, GPR50) [55] is the mammalian ortholog of Mel1c [56].

Using Chinese hamster ovary (CHO) cells [57,58] and the expression of the human MT1 (hMT1) in human embryonic kidney (HEK293) cells [59] several studies on heterologously expressed MT1 receptors have indicated a functional coupling of this isoform with cyclic adenosine monophosphate (cAMP) signal transduction via inhibitory heterotrimeric guanosine triphosphate (GTP)-binding proteins (Gi-proteins) and subsequent deactivation of protein kinase A (PKA). In addition, several researchers found compelling evidence that this receptor is also linked to the calcium signaling pathway via Gq/11 proteins and activation of phospholipase C (PLC) [60–62]. One report also indicated that melatonin inhibits protein kinase C (PKC) signaling [63]. In addition, evidence exists that this interface between the MT1 receptor and G-proteins is modulated via microtubuli rearrangements, and microtubuli depolymerization may explain phenomena like receptor desensitization [64]. Activation of a Gq-coupled pathway leads to a rapid and transient increase in intracellular Ca^2+^ according to the study by Brydon *et al.*[65]. An earlier study [60] using mouse fibroblast (NIH 3T3) cells as expression background for the hMT1 advocated the idea that the MT1 receptor maintains a certain amount of flexibility in its ability to couple with different G-protein α-subunits, namely with the pertussistoxin-sensitive Gi- or the insensitive Gq-protein. A single report [66], using heterologous expression of human MT1 or MT2 receptors in CHO cells, indicates the inhibitory potency of a coupling of both isoforms to the cAMP pathway, the PLC-dependent pathway and increased phosphoinositide (PI) hydrolysis. Inhibition of the phosphorylation of cAMP response element-binding protein (CREB) was shown to be the ultimate target of inhibitory melatonin signaling [67], leading to expression changes in a variety of CREB-susceptible genes containing a functional CRE consensus sequence. In a prostrate epithelial cell line, it has been reported that the antiproliferative action of melatonin is based on the dual activation of Gs- and Gq-proteins through the MT1 receptor [68].

Comparatively little is known about the signal transduction cascades of the MT2 receptor. This isoform has been proven to address both the cAMP and cGMP pathways via inhibitory G-proteins in a human preadipocyte cell line [69]. According to MacKenzie *et al.*[66], the MT2 is also coupled to PI hydrolysis.

Furthermore, melatonin activates the insulin receptor-mediated phosphoinositide 3-kinase (PIK3) and serine/threonine protein kinase B (PKB/Akt) and the mitogen-activated extracellular signal-regulated kinase kinase (MEK/ERK) pathway by inducing phosphorylation of the tyrosine moiety of the insulin receptor substrates 1 and 2 (IRS) in rat islets and INS-1 cells [70]. Melatonin also induces insulin receptor β-subunit phosphorylation in the rat hypothalamic *nucleus suprachiasmaticus* (SCN) via activation of its tyrosine kinase [71], which, in turn, triggers the mitogen-activated protein kinase (Akt/MAPK) pathway, indicating cross-talk between insulin and melatonin signaling. In the ovine *pars tuberalis* (PT), which displays a high level of MT1 receptors, melatonin inhibits phorbol 12,13 myristate acetate (PMA)-induced and PKC-dependent c-fos expression [63]. Via MT1, melatonin suppresses most clock genes in the PT, a central structure of circannual signaling and reproductive cycles [72]. Phase-shifting of circadian rhythms in slices of the SCN via MT2 also involves PKC activation [73]. An overview of the functional importance of melatonin receptors in peripheral tissues has been given in a recent review [74].

### 2.2. Receptor Heterodimerization

Whereas flexibility towards coupling to several G-proteins and thus signal transduction cascades (resulting in antagonistic effects on cells) presents one prominent feature of melatonin receptors, another layer of complexity was recently discovered in the heterodimerization of the isoforms. Although heterodimerization of normally homodimeric receptors is a well-known feature of GPRCs [75], heterodimerization of MT1 and MT2 isoforms has also become feasible using energy resonance transfer techniques (BRET) and MT1 or MT2 fusion protein co-expression [76]. A more recent paper [77] reports that heterodimerization has an even higher formation probability in their assay system than homodimerization of either receptor. Whether receptors of native tissues expressing both isoforms, particularly the pancreatic islet, form heterodimers still needs to be determined.

### 2.3. Receptor-Associated Proteins

The above-mentioned protein GPR50 has been found to be more than a mere evolutionary relict. Recent work [78] indicates that this protein may be involved in MT1 (but not MT2) receptor signal transduction. This conclusion stems from the observation that GPR50 negatively interferes in MT1, but not MT2, receptor signaling on the level of G-protein coupling. In addition, high-affinity ligand binding is inhibited. The authors propose that this mechanism functions through the formation of MT1/GPR50 heterodimers.

In recent years it has become clear that GPCRs, like melatonin receptors, all of which are anchored in the cellular membrane bilayer, can associate with other accessory proteins that affect signal transduction and receptor trafficking. Guillaume *et al.* describe the PSD-95/Drosophila Disc large/ZO-1 homology (PDZ) domain-containing protein (MUPP1) as a MT1-associated scaffold protein which itself is capable of interacting with a number of other proteins [79]. To give a more comprehensive overview, a list of MT1- and MT2-associated proteins in HEK293 cells has recently been published [80,81]. According to these data a picture emerges in which signaling via melatonin receptors involves a plethora of scaffold proteins in addition to G-proteins. Maurice *et al.*[82] identified no less than 22 proteins capable of interacting with the carboxy-terminal tail of MT1, and 14 proteins associated with MT2. Supporting these results, the interaction between one of the regulators of G-protein signaling (RGS20), which represents another group of multifunctional signaling proteins, and MT1 has been proven [83]. Thus, GPCR-coupled signaling seems to be a highly regulated process, possibly involving several dozen accessory proteins in a complex which may vary according to the cellular context in which these proteins are expressed. The pharmacology of MT1 and MT2 receptors (the latter only recently studied in mouse [84]) has been dealt with in several excellent reviews [85,86] and will not be discussed here.

### 2.4. Sensitization and Desensitization

Treatment of cultured cells with melatonin over prolonged periods, followed by rapid withdrawal, leads to enhanced cAMP signaling and CREB phosphorylation [67] after either forskolin stimulation (homologous sensitization, via Gi) or other ligand-activated receptor Gs-protein-coupled pathways (heterologous sensitization [87,88]) and is in stark contrast with the acute inhibitory effect of melatonin. This experimental design follows the natural exposure of receptors to melatonin during the night (typically 30–400 pM melatonin [85]), followed by a rapid decline of the hormone at dusk. Thus, sensitization may also be of relevance *in vivo*[89]. The exact mechanism of sensitization is as yet unknown and does not occur in all types of cells [89]. One report [67] proposed an increase in the undissociated state of Gi-proteins (heterotrimeric state) as a possible mechanism. Tyrosine phosphorylation of adenylate cyclases has also been suggested as a basis for sensitization processes in ovine PT primary cells [87]. As an *in vivo* phenomenon, the heterologous sensitization of melatonin signaling through concomitant activation of the adenosine A2b receptor in the PT of mice has been reported [88], a phenomenon thought to be unlikely to function, however, in ovine PT [89]. In contrast, *in vitro* experiments on CHO cells expressing MT1 or MT2 receptors indicated that chronic melatonin exposure leads to a loss of PI hydrolysis and attenuation of melatonin-mediated inhibition of (forskolin-stimulated) cAMP [66].

Melatonin receptors are also prone to desensitization mechanisms after chronic exposure to the indoleamine, which generally function in GPCRs by receptor internalization or uncoupling from G-protein subunits. The loss of receptor affinity or a reduction in receptor density has also been discussed [66]. One mechanism of desensitization, observed in CHO cells expressing recombinant hMT2, is downregulation by internalization [90]. The same authors reported that melatonin pretreatment in physiological amounts in SCN2.2 cells heterologously expressing the hMT2 leads to downregulation of PKC activity by receptor internalization and desensitization phenomena. According to Jarzynka *et al.*[64] desensitization in CHO cells expressing the hMT1 can be suppressed by colcemid treatment which suppresses microtubule depolymerization. The same authors suggest that hMT1 and Gi-, but not hMT1 and Gq-coupling, is affected by this mechanism. Another paper [91] also supports the idea that desensitization is linked to modulation of microtubuli aggregation and MT1 receptor internalization, as well as a change in cell morphology. Some data indicate that downregulation of the hMT1 is dependent on receptor density and/or constitutive activity [92]. Examination of the effects of chronic melatonin treatment on CHO cells expressing the hMT2 led to the discovery that particularly this isoform is downregulated after incubation over a physiological time span and with physiological melatonin concentrations [90]. In addition, the hMT2, heterologously expressed in SCN2.2 cells, is internalized after such pretreatment, decreasing its presence on the cell surface. The same authors have speculated that, *in vivo*, the nightly melatonin surge desensitizes endogenous MT2 receptors of the SCN, leading to a temporal shut down of the clock system’s sensitivity towards melatonin [90].

### 2.5. Function and Impact of Melatonin Receptors of the Pancreatic β-Cell on Insulin Secretion and Its Role within the Framework of Peripheral Circadian Clocks

The circadian clock is a three-part structure consisting of input signals, a clock or pacemaker and output by a rhythm generator [93]. Since the clock generates rhythms of *circa* (but not quite) 24 h, it needs constant readjustment (entrainment, e.g., light in the case of the self-sustained central pacemaker in the SCN). The clock generates circadian information on a transcriptional/translational level that is carried outward. The clock mechanism itself consists of rhythmically expressed genes (e.g., *Bmal1* and *Clock*, with stimulatory action, and *Per1* and *Cry1*, with inhibitory action), the products of which function as antiphasic heterodimeric transcription factors generating and perpetuating a circadian rhythm [93]. The clock mechanism works in organs as well as in single cells [94].

Early evidence showed that, besides the central circadian clock (master clock) in the SCN, peripheral clocks exist which are believed to be controlled by the master clock. However, unlike the master clock, these second-order clocks are entrained by metabolic cues (e.g., feeding [95]). Peschke and Peschke [96] and others [97,98], studying insulin secretion from isolated rat pancreatic islets, detected a circadian secretion pattern which is likely driven by an autonomous or semi-autonomous circadian clock. This can be assumed because rhythmic secretion from rat islets can be monitored in perifusion over a period of several days [96]. These data also complement the observed circadian fluctuation of insulin levels in human plasma [37,99]. This observation does not rule out the influence of the central circadian clock of the SCN. Such a link might function through neuronal sympathetic and parasympathetic connections [100,101].

According to the idea of an autonomous, islet-located clock, analysis of the circadian expression of clock genes was examined and proven on a transcriptional level in rat pancreas [102] and, more recently, by microarray analysis in human islets [103]. More than 12 years after the first evidence of an autonomous islet-located clock [96], a recent study [104] monitored the functioning of a self-sustained circadian pancreatic clock using a mouse model with a Per2-luc knockin-construct and recorded the circadian change of luciferase-derived chemoluminescence over 72 h. Although a gradual dampening of the cycles was monitored over time, the rhythm could be rescued by forskolin treatment of the islets. In the same study evidence of a close link between the proper functioning of an islet-located circadian clock and the development of type 2 diabetes, after conditional knockout of CLOCK or BMAL1 in islets, was provided. The authors reported altered islet size and architecture in *Clock*-mutant mice, together with altered expression of crucial islet genes and secretion defects, as a likely basis for the diabetic state. *Bmal1* conditional knockout mice, in contrast, displayed increased glucose levels and decreased insulin secretion.

In addition to recording circadian insulin secretion from isolated rat islets in perifusion [96], data from the same study also showed that melatonin causes a phase-shift in the circadian release of insulin. Shortly thereafter this line of research was complimented by the detection of melatonin receptor transcripts in islets of rodent and human pancreas [52,105], and was followed up by immunohistochemical evidence [106]. Taken together, melatonin seems to be important in synchronising the autonomous circadian clock of islet cells.

Whereas the expression of the MT1 isoform in islets was established quite early on both transcriptional [52,105] and translational levels (e.g., specific 125-I-melatonin binding on islets [51]), the expression of the MT2 became evident much later, due to the low level encountered [106–111]. Single-cell RT-PCR [112] failed to detect MT1 transcripts in isolated human β-cells, but succeeded in finding this isoform in α-cells. Which MT receptor isoform is functionally expressed in which of the major endocrine islet cells (α, β, or δ) is still a matter of debate, complicated by some findings which indicate species differences [112]. An immunohistochemical study [110], making use of a melatonin receptor knockout mouse model [113,114], revealed differential expression of MT receptors in islet cells. According to immunohistochemical data [110], MT1 receptors in mice are exclusively expressed in α-cells, whereas β-cells are endowed with MT2 receptors only. This finding contradicts the publication of Lyssenko *et al.*[108]. The latter authors reported that in human, rat and mouse islets, MT1 receptors can be detected in β-cells together with MT2 receptors, however, the former are less abundant.

There is also evidence for a divergence of receptor function between humans and rodents. Particularly notable was the observation that melatonin elicits an increase in insulin secretion from isolated human islets [112], whereas other authors found a reduction of insulin secretion in rodent islets [52,105,115]. A rise in Ca^2+^ in human islets has been linked to increased glucagon release from α-cells during incubation with 10 nM melatonin [112]; this was speculated to represent a paracrine reaction due to increased β-cell stimulation by glucagon. One has to keep in mind that in nocturnal species like rat, the peak melatonin level in the blood coincides with peak activity, whereas in man the maximum amount of melatonin released into the blood stream coincides with the resting and sleeping period. Disturbances of sleep leading to disruption of melatonin synthesis have therefore been linked to pathologically altered insulin secretion and diabetes [116,117].

Using the INS-1 rat insulinoma cell line, Kemp *et al.*[52] were among the first to prove that melatonin is a short-term inhibitory agent modulating glucagon-like peptide 1 (GLP1)-stimulated insulin secretion from pancreatic β-cells. Since these authors detected the MT1 receptor transcript by RT-PCR only, the observed and CRE-mediated inhibition of reporter gene activity was traced back to activation of Gi-coupled MT1 receptors and a subsequent modulation of the second messenger cAMP. In the same year, the group of Peschke [105] confirmed the inhibitory, Gi-mediated role of melatonin on forskolin-stimulated insulin secretion in the same cell model (INS-1). In addition, the crucial impact of cAMP, as a mediator of the melatonin response on insulin release, was proven in perifusion experiments during co-incubation with the unspecific phosphodiesterase-blocker 3-isobutyl-1-methylxanthine (IBMX) or with a cAMP-extrusion blocker (probenecid). Both pharmaceuticals lead to increased intracellular cAMP (and cGMP) levels. In rodents, the cAMP acts on the insulin secretion machinery via activation of Exchange Protein Activated by cAMP (EPAC); cAMP-regulated Guanine Nucleotide Exchange Factor-II (cAMP-GEFII) within the secretory complex [118] and/or via activation of PKA and phosphorylation of voltage-gated Ca^2+^ channels [119], although the former pathway is in doubt for human islets [118]. The activation of Ca^2+^ channels is at the end of the chain of events during glucose-stimulated insulin release (also known as stimulus-secretion coupling) [120]. The results of a study on INS-1 cells [52] also suggested that heterologous sensitization involving MT1 melatonin and GLP1 (the latter Gs-coupled) receptors may play a role in modulating insulin secretion in pancreatic β-cells after chronic melatonin exposure. This phenomenon has recently been confirmed [121] by a MT1-knockdown approach highlighting the relevance of this isoform in the mediation of sensitization effects on insulin secretion.

Whereas some data have been collected concerning melatonin signaling via MT1 receptors in islets and β-cells, the functional importance and signaling of the MT2 receptor still remains largely enigmatic. Stumpf *et al.*[122,123] were the first to prove that melatonin inhibits the cGMP pathway in INS-1 rat insulinoma β-cells. This process involves modulation of protein kinase G (PKG) activity via soluble guanylate cyclases (sGCs), which is a known feature of MT2 receptors [69]. A recent study [124] analyzed MT2 signaling in genetically modified INS-1 cells overexpressing the human MT2 (hMT2). Furthermore, the authors confirmed the inhibitory, acute action of melatonin on insulin secretion, which, in their cellular system, was largely due to MT2-driven reduction of cAMP and cGMP levels, with a negative impact on insulin secretion. Knockdown of the MT1 receptor in INS-1 cells significantly reduced the inhibitory effect of melatonin and indicated that, at least in rodent β-cells and islets, the majority of melatonin-elicited effects are transmitted via the MT1 isoform [121]. At the end of the cAMP signaling cascade, melatonin also negatively modulates CREB phosphorylation in INS-1 cells [125]. Activation of CREB has a consecutive impact on a variety of CRE-regulated genes, among them calcium/calmodulin-dependent protein kinase type II (CamK2d), as well as on glucagon and insulin expression [126–128]. Since debate continues concerning which of the two isoforms is expressed in human islets, as outlined above, activation of the MT2 receptor, which is predominantly expressed in human β-cells [108], may be of major importance *in vivo*.

### 2.6. Implications of Genetic Association Studies

Several genome-wide association studies in large cohorts of individuals with varying genetic background have revealed diabetes-susceptibility loci. One of the loci with a high and reproducible association with impaired insulin secretion and increased fasting glucose levels is represented by the gene for the MT2 receptor (*MTNR1B*), or, more specifically, by some of the common genetic variants (single nucleotide polymorphisms, SNPs) thereof [108,109,111,129–131]. The human *MTNR1B* gene consists of two exons, separated by approximately 9 kb of intronic sequence [54]. The risk G-allele rs10830963 of this gene was found to be linked to type 2 diabetes. Genome analysis has also shown that homozygous carriers of the risk allele display, among other traits, an impaired insulin response to oral glucose challenge [108], a reduced first-phase response during intravenous glucose challenge and reduced basal insulin secretion. Interestingly, the expression level of MT2 was raised in the islets of these individuals, which supports earlier observations [106] of the pancreata of type 2 diabetes patients in which MT1 as well as MT2 receptor expression was significantly increased. Recent data [132] on the risk allele rs10830963 in a Norwegian cohort failed to show a significant influence of this *MTNR1B* gene variant on the well-known association between sleep disturbances and type 2 diabetes.

**Figure 1:**
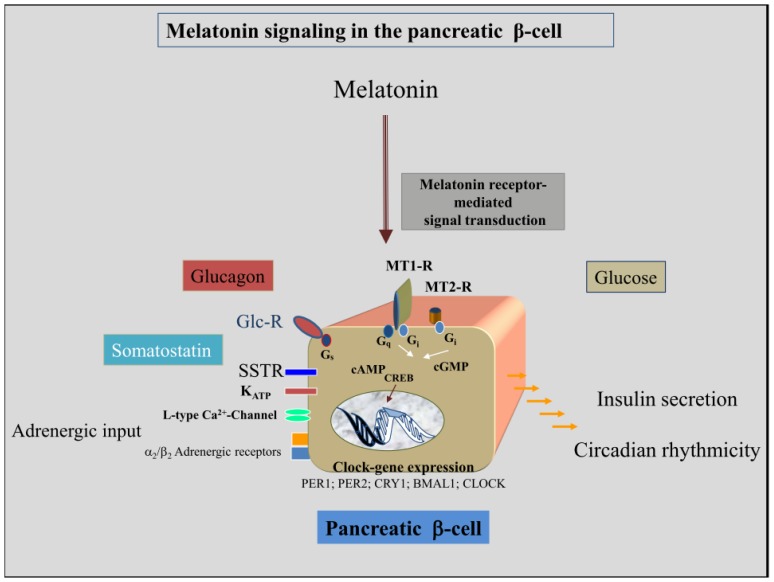
The pineal hormone melatonin acts on the pancreatic β-cell via two receptor isoforms (MT1 and MT2) which transmit their signals through guanosine triphosphate (GTP)-binding proteins (G-proteins). The inhibitory action of melatonin on insulin secretion is transmitted by activation of the Gi-protein signaling cascade involving cyclic adenosine monophosphate (cAMP) (MT1-dependent signaling) or cAMP and cyclic guanosine monophosphate (cGMP) as second messengers (MT2). Melatonin downregulates adenylate or guanylate cyclase activity, leading to reduced second messenger levels and attenuated protein kinase A (pKA) or protein kinase G (pKG) activity. Consecutively insulin secretion is reduced. As a secondary effect, phosphorylation and activation of the cAMP-modulated transcription factor cAMP response element-binding protein (CREB) is downregulated. In addition, the MT1 receptor is also known to alternatively couple to Gq-proteins and thus modulates cell-internal IP3 and Ca^2+^ levels. Melatonin signaling impinges on the rhythm of an autonomous, islet-located circadian clock as a synchronizer. This rhythm is generated by the antiphasic action of the clock genes *Per* and *Cry* (coding for a heterodimer with inhibitory action) or *Bmal* and *Clock* (producing heterodimeric proteins with transcriptionally enhancing action).

Since all of the identified common diabetes risk loci of the *MTNR1B* gene are outside of the coding sequences, the mechanism controlling how genetic variation affects insulin secretion and glucose homeostasis remains poorly understood. One study [133] attempted to find a link between diabetes and some of the rare genetic variants of the MT2 receptor that lie within coding regions of the gene. In functional expression studies on some of the gene variants, using monkey fibroblast-like kidney (COS-7) cells and altered signaling via a chimeric Gq-protein to monitor IP3 turnover, the authors succeeded in finding an association between some of the gene variants and impaired melatonin receptor signaling. However, there was no correlation between reductions in the melatonin signal and an increased type 2 diabetes risk or higher levels of fasting plasma glucose, even when signal transduction it totally disrupted. A recent study on a European cohort identified 40 gene variants with rare or very low-frequency distributions [134]. These variants within coding sequences of the *MTNR1B* gene were all non-synonymous (*i.e.*, leading to amino acid changes). Functional analyses by melatonin saturation binding experiments or by activation of the ERK1/2 pathway in human HEK293 cells indicated impairments in melatonin binding or receptor signal transmission via Gi-proteins. Some of these rare gene variants even displayed a total loss of function. This group of mutants displayed a clear association with type 2 diabetes, whereas another group of mutants with a partial loss of function were shown to have a higher risk of incurring diabetes. In summary, a close link between the functional impairment of *MTNR1B* gene variations and type 2 diabetes has been found. This stands in contrast with the results of Andersson *et al.*[133], who were unable to connect the loss-of-function of their tested gene mutants with a higher risk of diabetes. The above studies addressed rare mutations within exon sequences and with impacts on the amino acid composition of the receptor. Earlier results, however, on more common gene variants (which were all located outside coding sequences), and showing an influence on type 2 diabetes risk, still await functional explanation [108,111]. As a potentially disease-related effect (under conditions of decreased melatonin plasma concentrations [135]), the mRNA transcript levels of melatonin receptors appear to be significantly higher in type 2 diabetic patients than in a normoglycemic control group [106]. An upregulation of receptor expression in type 2 diabetic patients has also been observed in immunocytochemical investigations. Thus, the data demonstrate the existence of the melatonin membrane receptors MT1 and MT2 in human pancreatic tissue and that these receptors show upregulated expression levels in type 2 diabetic patients [106]. These results are in accordance with the fact that increased MT2 receptor mRNA is found in islets of individuals carrying the type 2 diabetes risk allele [108]. For functional interrelationships see Figure 1.

## 3. Recent Investigations of Melatonin on Insulin Secretion

Recently, using new techniques and methods and considering biorhythmic aspects like diurnal rhythms, it has been confirmed that the application of melatonin decreases insulin secretion. Without exception high levels of insulin are measured in rats when melatonin levels are reduced (during the day) and low levels of insulin together with high glucose levels are measured during the night when melatonin levels are increased [136,137]. In line with the cited results, studies in rats have shown that, with increasing age, the synthesis of melatonin declines, whereas the synthesis of insulin increases [30]. However, substitution of melatonin also appears to be able to stop the age-related increase of insulin [32]. Other publications have reported that melatonin levels are reduced in diabetic hamsters [35,36]. But again, evidence exists that substitution of melatonin helps to prevent diabetes. On the other hand, pinealectomy increases the risk [20,21].

The influence of melatonin on insulin secretion has been examined using an efficient dynamic perfusion system [138]. This study was conceived to investigate the specific function of melatonin on the controlled insulin secretion from explanted islets. In such experiments, administration of melatonin in both multiple short-term and single long-term manner does not alter the basal insulin secretion level; however, the stimulated insulin secretion by a specific (glucose) or nonspecific (KCl) stimulus is significantly reduced. This effect is reversible and repeatable. The data show that melatonin has a pronounced and specific effect on insulin secretion of isolated pancreatic islets. These results have been reproduced several times [51,105] and confirmed by others [52,115]. However, these studies opened a second line of inquiry: Are the described effects specific and are pancreatic β-cells endowed with melatonin receptors, as detected by Reppert and co-workers [53,54,139] in other cell types and tissues?

## 4. Melatonin-Receptor-Mediated Signal Transduction Pathways of the Insulin-Producing β-Cell

Detailed analyses of the melatonin-receptor-mediated intracellular signal transduction pathways are essential in order to better understand the functioning of the β-cell. In this context, the fact that melatonin exerts some of its biological effects on other organs through specific, high-affinity, pertussis toxin (PTX)-sensitive, Gi-protein-coupled receptors has been helpful [53,54,139]. Melatonin-receptor- coupled signal transduction is mediated by adenylate cyclases (AC) and, subsequently, by second messenger cyclic adenosine monophosphate (cAMP) [60,140,141]. It has also been shown that melatonin reduces forskolin-stimulated cAMP production, as well as insulin secretion, from isolated pancreatic islets of neonate rats and INS-1 cells [52,105,115,142,143]. As shown earlier, the competitive melatonin-receptor antagonist luzindole can mostly reverse the inhibitory effect of melatonin on AC/cAMP and insulin production [105]. In confirmation of these findings, the Giα-protein-inhibitor PTX abolishes the effect of melatonin on the levels of both cAMP and insulin. Taken together, these results clearly indicate that melatonin has an inhibitory influence on the cAMP-signaling pathway of the pancreatic β-cells, mediated via Giα-protein-coupled MT1 receptors. However, the intracellular signaling of melatonin in pancreatic β-cells is not limited to the cAMP-signaling pathway. Recently, it was discovered that melatonin also inhibits the guanylatcyclase/cyclic guanosine monophosphate (GC/cGMP) pathway [122,123]. Furthermore, evidence is accumulating for the involvement of the phospholipase C/1,4,5-trisphosphate (PLC/IP3) system in the signaling cascade of melatonin in a growing number of cell types. In contrast to the uniform cAMP- and cGMP-inhibiting effects of melatonin, both IP3-increasing [66,144] and IP3-decreasing [145–147] effects of melatonin have been described in different cell types. Previous results, obtained by IP3-mass assay on extracts from INS-1 cell-culture batches, have indicated dose-dependent stimulation of IP3 release by melatonin [148,149]. The type of MT1-receptor-coupled, G-protein-subunit-stimulating PLC can only be hypothesized.

Melatonin receptors expressed *in vitro* exhibit differential abilities for stimulating PLC via Gqα-proteins [150,151], and the MT1 receptor has been seen to couple with Gqα-proteins in an agonist-dependent and guanine-nucleotide-sensitive manner in HEK293 cells [61]. After selective inhibition of the cAMP-signaling pathway via PTX, a stimulatory effect of melatonin on carbachol-and even forskolin-stimulated insulin release occurs, probably due to activation of the IP3-signaling pathway by melatonin. As shown in earlier fluorescence-imaging studies, melatonin-induced IP3 liberation can mobilize intracellular Ca^2+^ concentrations [148], a mechanism that is commonly accepted as a trigger for insulin release. PTX has proven to be a valuable tool for distinguishing between the Giα-dependent cAMP and Giα-independent IP3 pathways. The successful inhibition of Giα-proteins was validated in each perfusion with rat INS-1 cells that included PTX. The inhibitory influence of melatonin on the cAMP-signaling pathway requires a longer incubation period; in contrast, the stimulatory influence of melatonin on IP3 levels is an instant effect [148]. Comparable effects of pineal hormone fractions have also been described, *i.e.*, first an increase followed by a decrease in insulin levels. Since IP3 is stimulated and cAMP is inhibited by melatonin, which would normally be conflicting signals for insulin secretion, the overall effect on insulin secretion was initially unclear. The perfusion results cited previously indicate a predominance of the cAMP- and, subsequently, the insulin-inhibiting pathway for melatonin. These findings confirmed earlier results in INS-1 cells [52,105] and whole islets [51].

In conclusion, melatonin receptors on pancreatic β-cells are coupled to three parallel signaling pathways, with differing influences on insulin secretion. In terms of insulin release, the AC/cAMP-pathway is dominant, leading to the inhibition of insulin secretion. Furthermore, possibly mediated by the MT2 receptor, melatonin inhibits the guanylatcyclase/cyclic guanosine monophosphate (GC/cGMP) pathway and, consecutively, also inhibits insulin secretion. Melatonin-mediated IP3-release may play a role in the short-term support of other IP3-releasing agents, like acetylcholine; it may also be related to the activation of PKC or to the long-term regulation of β-cell functions, both with stimulatory effects on insulin secretion. Thus, the influence of melatonin on pancreatic β-cells and on insulin secretion is connected with a complex pattern of intracellular signal transduction pathways, including the cAMP-, cGMP- and IP3-signaling pathways (for more information see earlier reviews [152,153]).

## 5. Biological Relevance of Melatonin-Insulin Antagonisms in Metabolic Disturbances, as well as in Type 1 and Type 2 Diabetes

The wide spectrum of biological activities involving melatonin, e.g., the circadian organization of physiological functions, the evidence that melatonin stabilizes and strengthens the coupling of circadian rhythms [154], and, particularly, its main characteristic as a regulator of biological rhythms, indicate that it is an important regulator for the coordination of intercellular interactions [155]. Reductions in melatonin secretion by pinealectomy or sympathetic denervation of the upper sympathetic ganglia have been associated with disturbances in the circannual rhythms of body weight and food intake [156], as well as in many other metabolic disorders, for example, diabetes [106,135]. These observations indicate that the diurnal melatonin signal is essential for glucose homeostasis and regulation [154].

In humans, circulating melatonin shows a circadian rhythm, peaking at night. The rhythm-adjusted mean (mesor) is higher in women than in men, which has been observed particularly in elderly women [157]. The circadian amplitude decreases with age and may be regarded as a marker of the aging process itself [158]. The reduction in melatonin with age may be a factor of increased oxidative damage in the elderly [159], including age-associated neurodegenerative diseases [160]. The mesor and the amplitude of melatonin are modulated by annual variation, the mesor peaking in winter and the amplitude in summer [161]. In nightshift workers, insulin, glucose and triglycerol are higher after a nighttime meal than after a daytime meal. These abnormal values are an expression of the de-synchronization of bodily functions, leading to a higher incidence of heart diseases [162] and metabolic disturbances like diabetes. To emphasize this point, it should be mentioned that a nocturnal lifestyle is likely one of the major health risks to modern man, including night-eating syndrome, obesity and diabetes [163].

Like melatonin, insulin levels show a circadian rhythm in *in vitro* experiments [96], as well as in humans; the rhythm is, however, opposite that of melatonin, *i.e.*, insulin is at a maximum when melatonin is at its lowest level and *vice versa*[37]. In this context, a recent paper is important which demonstrated that long-term enteral administration of melatonin reduces plasma insulin and increases expression of pineal insulin receptors in both healthy Wistar rats, as well as type 2 diabetic Goto-Kakizaki (GK) rats [164], whereas pineal melatonin synthesis is decreased in type 2 diabetic GK rats [165]. These results are in agreement with recent *in vitro* investigations of pancreatic islets [51,96,138], as well as with studies of the rat insulinoma cells INS-1 [105,166] described above, namely that plasma insulin levels are diminished after melatonin treatment.

**Figure 2:**
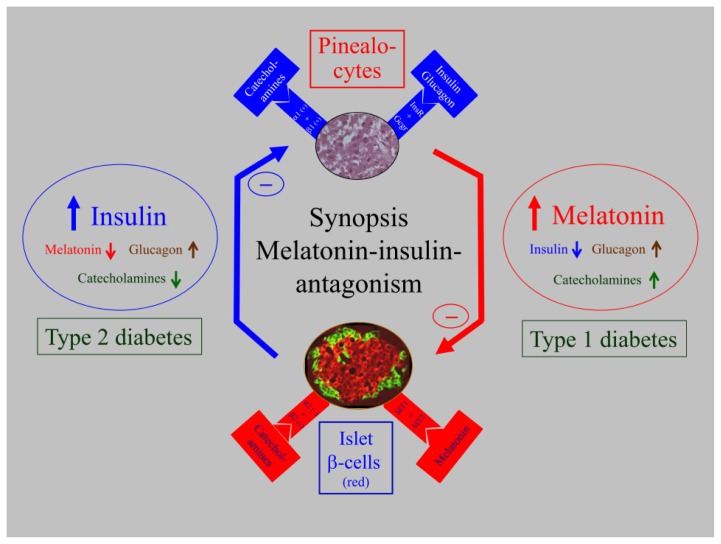
Synoptic presentation of the insulin–melatonin antagonism in relation to the importance of melatonin for type 1 and type 2 diabetes, including interactions with glucagon and catecholamines. At a relatively early stage of type 2 diabetes (left side), insulin secretion is increased while melatonin synthesis is decreased. These reactions were observed in type 2 diabetic Goto Kakizaki (GK) rats and humans. In contrast, under type 1 diabetic conditions (right side), insulin was greatly reduced and, subsequently, melatonin was significantly increased. These reactions were observed in streptozotocin (STZ)-treated Wistar (WR) rats, as well as in LEW.1AR1-*iddm* rats, a spontaneous animal model of human type 1 diabetes mellitus. Thus, the influence of insulin on the pinealocytes is mediated by insulin receptors in the pineal gland (upper panel), and the influence of melatonin on the pancreatic β-cells is mediated by the MT1 and MT2 melatonin receptors (lower panel). Much more conclusive, however, is the well-known insulin-catecholamine relationship, which may be a key to understanding the insulin-melatonin antagonism. The increase of catecholamines stimulates adrenoceptor β1 and, consequently, the cAMP cascade, while adrenoceptor α1 activates the IP3 cascade in the pineal gland; together they stimulate melatonin synthesis and secretion (upper panel). Catecholamines (in contrast to acetylcholine), on the other hand, have an inhibitory effect on the insulin secretion (lower panel). To support this, the GK rat model of type 2 diabetes shows diminished plasma catecholamines (left side), whereas the rat models of type 1 diabetes (STZ and LEW.1AR1-*iddm*) exhibit increased catecholamines (right side). This supports the conviction that type 1 diabetes is associated with stress and enhanced melatonin secretion. An explanation of the increased melatonin levels in type 1 diabetic STZ and also possibly LEW.1AR1-*iddm* rats could be that melatonin protects the organism by attenuating the oxidative stress-induced β-cell damage in type 1 diabetes.

Reports on the interactions between melatonin, glucose metabolism of rats and humans have shown phenomenological and functional causal-analytic results. Molecular and immunocytochemical investigations established the presence of the melatonin receptors MT1 and MT2 in human pancreatic tissue [106] and, notably, also in rat islets [107]. Results of a calculation model to determine mRNA expression ratios demonstrated elevated MT1 receptor expression in comparison to MT2 expression. The mRNA transcript levels of melatonin receptors appeared to be significantly higher in type 2 diabetic patients than in a control group. An upregulation of receptor expression in type 2 diabetic patients has also been observed in immunocytochemical investigations [106]. This upregulation is combined with both lower plasma melatonin levels and reduced arylalkylamine-N-acetyltransferase (AA-NAT) enzyme activity of the pineal gland (AA-NAT being the key enzyme in melatonin synthesis), whereas the AA-NAT-mRNA from rat pineal glands appears to be increased [135]. The insulin receptor mRNA of the pineal gland was found to be reduced in type 2 diabetic rats, suggesting a functional interrelationship between melatonin and insulin [135]. In this context, recent results are important which reported that melatonin-enhanced insulin-receptor kinase activity increases insulin-receptor substrate-1(IRS1) phosphorylation, suggesting the potential existence of signaling pathway cross-talk between melatonin and insulin, possibly also in the pinealocytes [152,167]. The insulin-melatonin interactions are summarized in a synoptic presentation (Figure 2, left side). In a relatively early stage of type 2 diabetes, insulin secretion is increased and melatonin is decreased—a pattern that is observed in rats and humans. It was hypothesized that catecholamines, especially norepinephrine, which decrease insulin levels and stimulate melatonin synthesis, trigger the antagonistic insulin-melatonin interactions [168,169].

In addition, the melatonin-insulin interaction in type 1 diabetes is of interest. Whereas type 2 diabetic rats and humans show slightly increased plasma levels of insulin, combined with clearly decreased melatonin levels, the situation in streptozotocin (STZ)-induced type 1 diabetic rats is completely different. Both 12 week old and 51 week old Wistar rats show extremely decreased levels of insulin and, surprisingly, increased plasma melatonin levels and elevated AA-NAT mRNA in the pineal gland. Furthermore, the mRNA levels of the pineal insulin receptors and β1-adrenoceptors, including the clock genes *Per1* and *Bmal1*, as well as the clock-controlled output gene *Dbp*, are increased in young and middle-aged STZ rats. Therefore, the results indicate that, in this animal model, the extremely decreased insulin levels in STZ-induced type 1 diabetes are associated with higher melatonin plasma levels (for more information see [170]). In summary, melatonin and insulin plasma levels in type 2 compared to type 1 diabetes are totally different. Although these results are in agreement with observations that administration of melatonin [30,31,34,171–173] and pinealectomy [14,174] affect metabolic disturbances related to plasma insulin and diabetes, doubts remain as to whether the described effects after STZ application can be generalized. This question is relevant because the systemic toxicity of STZ may cause lateral effects. To obtain unequivocal evidence of the relevance of the STZ-induced effects on insulin and melatonin, these parameters were analyzed in a spontaneous animal model of human type 1 diabetes, the LEW.1AR1-iddm rat. In addition the effect of insulin substitution on melatonin levels was investigated using implanted insulin pellets. The results were similar to those for STZ-treated rats. Severe hypoinsulinaemia in diabetic LEW.1AR1-iddm rats is associated with decreased body weight and increased melatonin plasma levels combined primarily with the elevated expression of *Aanat*, *Hiomt* (hydroxyindole-*O*-methyltransferase), the pineal insulin receptor and the adrenoceptor β1 in male and female rats These changes can be normalized by insulin substitution. The diurnal profiles of plasma melatonin and the antagonistic clock genes *Per1* and *Bmal1* are maintained in diabetic and insulin-substituted rats (for more information see the right side of Figure 2 and [175]).

Finally, what is the biological relevance of the observations and what controls the described reactions? It has been hypothesized that catecholamines, which decrease insulin levels and stimulate melatonin synthesis, control insulin-melatonin interactions. Thus, it is important to note that norepinephrine is the most decisive stimulator of melatonin synthesis, whereas epinephrine has an inhibitory effect on insulin secretion mediated through α2 receptors [176,177]. In contrast, through the activation of β2 receptors, which are Gs mediated, norepinephrine stimulates insulin secretion [176]. In this context, results are important which have shown that the inhibitory α2 receptors, which are localized on the presynaptic noradrenergic terminals of the pineal gland [141], are significantly more strongly expressed in GK than in Wistar rats [168]. Concerning noradrenergic α2 receptors, it is well known that, when these adrenergic receptors are expressed, noradrenaline can act either through the autoreceptors of the nerve terminals suppressing its release or through this receptor expressed on pinealocytes [141]. The signal transduction pathway may be through inhibitory Gi-proteins acting on the cAMP level [177,178]. Since expression analysis of adrenoceptor α2 was performed on the pineal gland, it is unlikely that relative expression data were confounded by transcripts from postsynaptic neuronal connections to the pineal gland. The increase in receptor expression observed in GK rat pineal glands implies a reduced cAMP level and, as a secondary effect, reduced melatonin synthesis, which is cAMP dependent [179]. This may explain the reduced noradrenergic stimulation reported and why the pineal glands of GK rats produce less melatonin as a reaction to norepinephrine [165]. Furthermore, inverse correlations between catecholamines and insulin have been described in recent investigations [169,175]. The sympathetic fibers connecting the pineal gland are derived from the *centrum ciliospinale* (segments cervical 8 to thoracic 2), switch in the upper cervical ganglia and reach the pineal gland as the *nervi conarii*. The liberated epinephrine vesicles of these nerve endings activate, via β1 receptors, the cAMP pathway and, concomitantly, activate, via α1 receptors, the IP3 cascade, thus raising the level of Ca^2+^. As a result, melatonin synthesis is activated in the gland [141,180–182]. Recent publications support this assertion; it has been shown that both catecholamines and melatonin plasma levels are enhanced in type 1 diabetes, but both are diminished in type 2 diabetes (see Figure 2 and [169]). Another important line of inquiry involves the fact that melatonin protects β-cells against functional overcharge and, consequently, hinders the development of type 2 diabetes. In this context, it is striking that, with advanced age, melatonin levels are reduced and the incidence of type 2 diabetes increases. Thus, melatonin appears to have a protective biological role. Here, we strongly repudiate misconceptions, resulting from observations that melatonin reduces the plasma insulin level, that the blockage of melatonin receptors would be of benefit in the treatment of type 2 diabetes. On the other hand, in the case of type 1 diabetes, increased melatonin levels could signify a protective reaction of the organism. As proposed by Reiter *et al.*[183], this reaction appears to counteract diabetes-induced stress, thereby attenuating oxidative stress-induced β-cell damage in type 1 diabetes, as previously suggested in another review [184].

## 6. Recent Investigations of Melatonin-Glucagon Interrelationships

As an antagonistic hormone for insulin, glucagon is one of the most important regulators of blood glucose homeostasis *in vivo*. Glucagon is secreted from pancreatic α-cells under hyperglycemic conditions and stimulates the hepatic glucose output. The hormone leads to a rise in blood glucose levels by increasing glycogenolysis and gluconeogenesis, as well as by decreasing glycogenesis and glycolysis via multiple mechanisms.

In addition to impaired pancreatic insulin secretion and peripheral insulin action, pathological α-cell physiology and glucagon release also play important roles in initiating and maintaining hyperglycemic states in diabetes. Under hypoglycemic conditions type 1 and type 2 diabetic patients show elevated ratios of glucagon and insulin, hyperglucagonemia, as well as an altered secretory response of α-cells [185–187]. These disturbances in α-cell physiology in diabetic states have not yet been thoroughly described; impaired glucose sensing in α-cells, autonomic neural dysfunction, insulin resistance and the loss of β-cell function have been discussed in this regard [188,189]. Several studies have reported a clear circadian rhythm of physiological glucagon secretion [190–192]. This daytime-dependent glucagon secretion is independent of feeding conditions and is governed by the central circadian clock, which is located in the hypothalamic SCN [193]. Examinations with pinealectomized rats have provided conflicting evidence on the influence of the synchronizing hormone melatonin—an important neuroendocrine output of the circadian clock—on glucagon secretion [14,194]. In addition, two studies by Schmid *et al.*[195,196] showed that both total sleep loss and sleep restriction are associated with reduced plasma glucagon levels in humans. These *in vivo* observations have led to the assumption that melatonin not only influences pancreatic insulin secretion, but also affects the glucagon secretion of islet α-cells.

To investigate the direct effect of melatonin on pancreatic α-cells, the mouse glucagon-producing α-cell line αTC1.9 was employed. This cell line has been used extensively to study the properties of pancreatic α-cells and has been found to be glucose responsive [197,198]. Molecular analyses have also revealed expression of both the melatonin receptors MT1 and MT2 in αTC1.9 cells [198]. Similarly, studies on human pancreatic islets have demonstrated that both melatonin receptors are expressed, although they are primarily detected in β-cells [108]. However, in single-cell PCR analyses on isolated human α-cells and in immunocytochemical investigations on murine islets, only the MT1, but not the MT2 receptor, is detected [110,112]. These dissimilar results may have been caused by low melatonin receptor expression levels, species-specific varieties, the poor performance of available antibodies, or the different turnover rates of melatonin receptors with low mRNA and high protein levels [110].

Incubation experiments with αTC1.9 cells clearly demonstrated that melatonin in physiological and pharmacological concentrations increased the expression and secretion of glucagon [198]. Hence, a direct influence of melatonin on pancreatic α-cells was revealed for the first time. These results have been confirmed in isolated islets of Langerhans: Melatonin incubation leads to enhanced glucagon release in islets of metabolically healthy Wistar rats, as well as in islets of type 2 diabetic GK rats [199]. In accordance, perifusion studies on isolated human pancreatic islets demonstrated that melatonin application induces an increase in glucagon secretion [112]. Therefore, it was possible to establish the glucagon-increasing action of melatonin in cell assemblies of the islets of Langerhans, where paracrine effects of adjacent β, δ, or PP (PP, pancreatic polypeptide) cells may also modulate the glucagon secretion of α-cells. An indirect melatonin effect on glucagon release via modulating insulin secretion of pancreatic β-cells has been discussed [112]. Although such interplay between islet cells cannot be ruled out, the above-mentioned results of incubation studies on αTC1.9 cells conclusively prove that melatonin has a direct effect on α-cells.

In addition to *in vitro* studies, investigations on rats and mice have verified the glucagon-increasing effect of melatonin: Long-term oral administration of melatonin over nine weeks resulted in significantly elevated plasma glucagon concentrations in Wistar rats compared to concentrations in the ethanol-treated control group [164,198]. Interestingly, this study further demonstrates hyperglucagonemia in type 2 diabetic GK rats, but plasma glucagon concentrations are slightly decreased by melatonin [198]. The lack of glucagon increase after melatonin treatment in GK rats may be a consequence of impaired insulin secretion or disturbed α-cell sensitivity for glucose, as is characteristic in diabetes [200,201]. However, incubation experiments with melatonin using isolated islets of GK rats resulted in enhanced glucagon release [199]. These discrepant observations may be ascribed to the different ages of animal material. The GK rat islets were isolated from neonate rats, and their physiology was obviously not yet disturbed. In contrast, the animals for long-term melatonin treatment were already eight weeks old when the experiments began, and characteristic diabetic disturbances of islet cell physiology were pronounced [202,203].

As the insulin-inhibiting effect of melatonin was shown to be receptor-mediated; the question arose as to whether melatonin regulates α-cell glucagon secretion via the melatonin receptors as well. Incubation experiments with αTC1.9 cells using the nonselective melatonin receptor antagonist luzindole; as well as the MT2 receptor-specific antagonist 4P-PDOT; provided evidence for the first time that melatonin mediates its effects on glucagon secretion via melatonin receptors [199]. In addition; investigations on islets of Langerhans isolated from wild-type mice and melatonin receptor knockout mice confirmed these results: Compared to islets of the wild-type control group; basal glucagon release was significantly decreased in the islets of mice with their MT2 receptor or both melatonin receptors knocked out [199]. Moreover, *in vivo* experiments in the above study demonstrated that plasma glucagon concentrations were also reduced in mice lacking the melatonin receptors. Both the cell incubation studies with melatonin receptor antagonists and the decreased glucagon secretion in MT2 receptor knockout mice—but not in MT1 receptor knockout mice—indicate the dominance of MT2 receptor signaling in mediating melatonin effects in pancreatic α-cells [199]. These results are of great interest as several genome-wide association studies have demonstrated that genetic variations in MT1 and MT2 melatonin receptors are associated with disturbances in glucose metabolism [108,109,111,129,204]. During the past few years; the receptor-mediated inhibition of insulin action by melatonin has provided an explanation for this association. With the knowledge that melatonin induces its effects on glucagon secretion—also via melatonin receptors—alterations in glucose metabolism brought about by MT1 or MT2 receptor polymorphisms may also be caused by melatonin action on pancreatic α-cells. However; further investigations; like binding studies or examinations of primary α-cells transfected with the MT1 or MT2 receptor; will be indispensable to fully clarify the function of melatonin receptors on glucagon-producing α-cells.

A second line of inquiry to obtain information on how melatonin functions in α-cells aims at determining which intracellular signaling cascade is involved in mediating the effects of melatonin. As already mentioned above, melatonin is known to act by modulating the concentrations of intracellular messengers like cAMP, cGMP, IP3, arachidonic acid, and calcium ions in various cell and tissue systems. Though, melatonin acts mostly in an inhibitory manner by downregulating AC activity via PTX-sensitive Gαi-proteins and consecutive reduction of cAMP concentrations [205], incubation studies on αTC1.9 cells have ruled out the involvement of the Gαi-coupled signaling pathway. Preincubation with PTX did not prevent the glucagon-increasing effect of melatonin, and cAMP concentrations were not affected by melatonin administration either [199]. Furthermore, the unaltered cAMP concentrations after melatonin incubation also ruled out the Gαs-signaling cascade, as modulation of this pathway would lead to changes in cAMP levels. In contrast, the inhibition of PLC in αTC1.9 cells reversed the glucagon-enhancing effect of melatonin, which indicates that the Gαq signaling pathway is involved [199]. Receptor-dependent melatonin actions mediated via Gαq-coupled proteins have previously been established in other cell systems, like prostate epithelial cells, breast cancer cells and pancreatic β-cells [68,148,206]. Furthermore, in hamster glucagonoma In-R1-G9 cells, PLC has already been shown to be involved in the intracellular regulation of glucagon secretion [207–209]. Thus, with respect to the action of melatonin in pancreatic α-cells, it appears that melatonin binds with melatonin receptors coupled to Gαq proteins, subsequently activating the PLC that hydrolyses phosphatidylinositol 4,5-bisphosphate (PIP2). This action leads, in turn, to release of the intracellular messengers IP3 and diacylglycerol (DAG). The IP3-induced calcium secretion from intracellular calcium stores is thus triggered, inducing an increase in glucagon secretion [210].

A second signaling mechanism involved in mediating the effects of melatonin in pancreatic α-cells is the modulation of phosphatidylinositol 3-kinase (PI3K). Incubation experiments with αTC1.9 cells have demonstrated that the glucagon-enhancing effect of melatonin can be prevented by co-incubation with the PI3K inhibitor wortmannin [199]. In earlier studies, melatonin has been shown to regulate growth, differentiation and survival of cells via PI3K in astrocytes, endothelial cells and even in rat pancreatic islets [70,211,212]. To what extent melatonin also influences these features in α-cells currently remains unclear. In addition, the possibility of cross-talks between the PLC and PI3K signaling pathways has to be considered for pancreatic β-cells, as described by Batty *et al.* for astrocytoma cells [213].

Thirdly, regulation of the glucagon promoter by melatonin-induced activation of PLC and an increase in intracellular calcium was taken into consideration, as melatonin requires long incubation times to significantly affect glucagon secretion [199]. Calcium influx stimulated glucagon gene transcription through dimerization of the transcription factors NFATp and HNF-3β [126] or by phosphorylation of the transcription factor CREB [214]. However, further studies will be required to specify the signaling cascades involved in mediating melatonin effects via melatonin receptors in pancreatic α-cells.

Investigations into the influence of melatonin on glucagon established not only a direct effect on pancreatic α-cells, but also on peripheral glucagon action in the liver [198,199]. Glucagon primarily acts on hepatocytes to stimulate the release of glucose and, therefore, to protect the organism against hypoglycemia. Interestingly, circadian variations in hepatic gluconeogenesis and glycogen storage have been detected [215–217]. It is well known that clock genes and clock output genes are rhythmically expressed, indicating the existence of a peripheral clock in the liver. It has been demonstrated that disruption of the hepatic clock components results in alterations of glucose homeostasis and the activity of gluconeogenesis [218,219]. Controlled by the SCN, humoral factors like glucocorticoids and melatonin synchronize the hepatic peripheral oscillations. *In vivo* studies have demonstrated that a knockout of melatonin receptors is associated with changes in the amplitude or maximum of the rhythmic expression of clock genes and clock-controlled output genes in the liver [220]. Binding studies have also provided evidence that melatonin modulates plasma glucose levels by binding to hepatic melatonin receptors [221]. In addition, a melatonin effect on glycogen synthesis and glucose utilization has been established in cell and *in vivo* investigations [222,223]. Notably, molecular analyses of the hepatic glucagon receptor in rats and mice have revealed that melatonin influences the expression of glucagon receptor mRNA levels in the liver: Compared to the wild-type control group, mice with knockouts of MT1, MT2, or both melatonin receptors are characterized by significantly higher glucagon receptor mRNA levels their liver [198]. In addition, studies on pinealectomized rats demonstrated an enhanced number of glucagon receptors and increased glucagon binding on liver membranes - an effect that could be reversed by melatonin administration [14]. In accordance to these results, oral treatment of WR rats with melatonin for nine weeks resulted in reduced hepatic expression of glucagon receptor mRNA [198]. Interestingly, in contrast to metabolically healthy WR rats, the hepatic glucagon receptor mRNA of type 2 diabetic GK rats was elevated [198]. Thus, an increase in plasma glucagon is associated with a decrease in the hepatic expression of glucagon receptor mRNA in Wistar rats. In contrast, GK rats reveal slightly reduced plasma glucagon concentrations and an enhanced glucagon receptor mRNA level. Consequently, it can be speculated that melatonin mediates its effects on hepatic glucose metabolism by varying the hepatic glucagon action via regulation of the glucagon receptor expression.

In conclusion, the existing data clearly show that melatonin has a stimulatory effect on glucagon secretion in pancreatic α-cells and on hepatic glucagon action in the liver, and indicate disturbances of these effects in diabetes. It is well known that disturbances in circadian rhythms caused by sleeping disorders or shift work are associated with a higher risk of impaired glucose tolerance and type 2 diabetes [224–226]. The altered influence of the synchronizing hormone melatonin on pancreatic glucagon release and the peripheral glucagon action in diabetic individuals provides a possible explanation for this association. With the knowledge that the effect of melatonin on α-cells is receptor mediated, modulation of glucagon secretion through synthetic analogues or melatonin receptor antagonists as therapeutic agents could be considered for the regulation of blood glucose homeostasis in diabetic patients. But until now, because of the different effects of melatonin on pancreatic islet cells, it remains unclear whether the administration of such substances would mediate anti-diabetic or even diabetic effects. Short- and long-term *in vivo* investigations using melatonin agonists and melatonin receptor inhibitors are absolutely necessary in the future to study the influence of melatonin on glucose metabolism. It is vital that the described effects of melatonin on insulin and glucagon secretion be considered when using melatonin, melatonin receptor inhibitors, or synthetic analogs as nutritional supplements, antidepressant agents, or in the treatment of sleeping disorders. Such substances are legal and widely used, but their impact on glucose metabolism after frequent or long-term consumption needs further investigation.

A complication faced when investigating the effects of melatonin is that the hormone is released during the night (the dark period) in all mammals, independent of their diurnal or nocturnal behavior. However, most experimental studies have been performed on rats or mice, *i.e.*, on nocturnal animals; thus, the transferability of results to humans is somewhat limited. However, a melatonin-mediated stimulation of glucagon secretion at night can be explained as a mechanism to provide energy during the active period, even during fasting. An increase in the release of glucagon in diurnal mammals may enable constant blood glucose concentrations during inactive periods without food intake. Furthermore, the glucagon-enhancing effect of melatonin could be established in rats, mice and human islets [112,198,199], which indicates that melatonin plays a role in maintaining blood glucose homeostasis in all mammals.

## 7. Conclusions

Two decades ago, the functional importance of melatonin was grossly underestimated. Recent advances in the elucidation of melatonin receptor expression, function and signaling have changed this situation dramatically. The data discussed in this review shed new light on melatonin as a hormone which acts on diverse physiological processes in mammals and, particularly, on pancreatic islet functions. General agreement exists that the physical effects of melatonin are mediated through the activity of two known membrane receptor isoforms, MT1 and MT2, which belong to the large family of G-protein-coupled receptors. Numerous studies support the notion that activation of either receptor isoform leads to a reduction in second messenger cAMP or, in the case of the MT2, also to cGMP levels, due to a coupling of the receptor with G-proteins which inhibit AC activity. Within endocrine cells, like the pancreatic β-cells, a reduction in cAMP levels is accompanied by reduced insulin secretion, at least in rodent β-cell lines and islets. In contrast, secretion of glucagon from α-cells is increased, which may indicate a coupling of the melatonin receptor with a different type of G-protein. Thus, the interplay between the different cell types of the pancreatic islet and their respective secretion products needs to be studied in more detail.

In mammals, insulin and melatonin are regulated in an inverse fashion. In a rat model of type 2 diabetes, insulin was found to be increased due to the well-known phenomenon of insulin resistance. Plasma melatonin levels, however, were below those of normoglycemic WR controls. In the rat model of type 1 diabetes, with chemically ablated β-cells due to STZ treatment, melatonin levels were strongly increased, whereas plasma insulin concentrations were generally below detection limit. This finding has been corroborated in a spontaneous model of type 1 diabetes, the IDDM rat, which, despite a different genesis of type 1 diabetes, also displayed the same characteristic inverse melatonin-insulin relationship. In summary, both models of type 1 and type 2 diabetes support the proposal that melatonin and insulin represent an antagonistic relationship.
